# Successful management of large conjunctival squamous cell carcinoma with excisional biopsy, topical interferon, and amniotic membrane grafting: A case report^☆^

**DOI:** 10.1016/j.ijscr.2025.110820

**Published:** 2025-01-07

**Authors:** Amirmohammad Fathian, Navid Fazlinejad, Alireza Attar

**Affiliations:** Poostchi Ophthalmology Research Center, Department of Ophthalmology, School of Medicine, Shiraz University of Medical Sciences, Shiraz, Iran

**Keywords:** Conjunctival squamous cell carcinoma, Excisional biopsy, Amniotic membrane graft

## Abstract

**Introduction and importance:**

Conjunctival squamous cell carcinoma (SCC) is the most advanced form of ocular surface squamous neoplasia (OSSN), with varying incidence rates influenced by factors such as age, UV exposure, and occupation. Early detection is crucial, but misdiagnosis is common, especially when SCC mimics benign conditions like pterygium.

**Case presentation:**

An 83-year-old Caucasian male farmer presented with a rapidly enlarging nasal limbal lesion, initially misdiagnosed as pterygium. Clinical evaluation suggested malignancy, and an excisional biopsy using the “No Touch” technique was performed to avoid tumor seeding. Histopathology confirmed conjunctival SCC, with 10 % of the lesion remaining post-excision due to its large size. Adjuvant treatment with topical Interferon alpha-2a (1 MIU/cc) targeted the residual tumor. Amniotic membrane grafting was employed to manage the wound, and topical Mitomycin C was used to reduce the risk of recurrence. Follow-ups at 1 month, 3 months, 6 months, and 1 year showed no signs of recurrence.

**Discussion:**

This case underscores the importance of distinguishing conjunctival SCC from benign lesions. The combined approach of surgical excision, topical immunotherapy, and chemoprophylaxis was effective in treating this advanced SCC case, preventing tumor recurrence.

**Conclusion:**

A multidisciplinary treatment strategy can effectively manage advanced conjunctival SCC, as seen in this case, where no recurrence occurred after one year of follow-up. Early detection and comprehensive care are essential for positive outcomes.

## Highlights

• Conjunctival squamous cell carcinoma (SCC) is a serious ocular surface malignancy often linked to UV exposure.
• An 83-year-old Caucasian male farmer with a large nasal limbal SCC was initially misdiagnosed as pterygium.
• Surgical excisional biopsy using the "No Touch" technique removed 90% of the lesion, while topical Interferon alpha-2a treated the remaining 10%.
• Amniotic membrane grafting and Mitomycin C were used to manage the surgical defect and reduce recurrence risk.
• After 1 year of follow-up, the patient remained tumor-free, highlighting the effectiveness of a multidisciplinary approach in managing advanced conjunctival SCC.

## 1 Introduction

Conjunctival squamous cell carcinoma is the extension of the dysplastic conjunctival epithelium through the basement membrane to reach the stroma [1]. The total incidence of conjunctival SCC was 0.503 per 1,000,000 population between 1975 and 2019 which was more prevalent in men rather than women; a significant difference was seen regarding race which means white people accounted for more SCC in comparison to other races [1]. Environmental factors such as ultraviolet light exposure are a risk factor for developing SCC [2]. Tumor size is divided into three categories: small, medium, and large tumors. First of which was defined as tumors less than 3 clock hours for limbal ones and if the largest diameter of tumor was less than 5 mm. medium size tumors were defined as those 3 to 6 h for limbal ones or if the largest diameter was between 5 and 10 mm, and large tumors were defined as those more than 6 clock hours for limbal ones or if the largest diameter was more than 10 mm [3]. Based on the extension of the lesion different kinds of management are available. In general, alcohol epitheliectomy for the corneal component and partial lamellar scleroconjunctivectomy with wide margins for the conjunctival component are used in limbal lesions which are followed by cryotherapy. Microscopically controlled excision can be done to ensure a tumor-free margin. The other method is wide local resection plus cryotherapy for forniceal tumors. A mucous membrane graft or amniotic membrane graft may be used in case of excessive conjunctival loss [4]. The purpose of this study is to illustrate effective management strategies for advanced conjunctival squamous cell carcinoma using combined surgical and pharmacological treatments.

Our work has been reported in line with the SCARE Guidelines 2023 criteria [5].

## 2 Case report

**Fig. 1:**
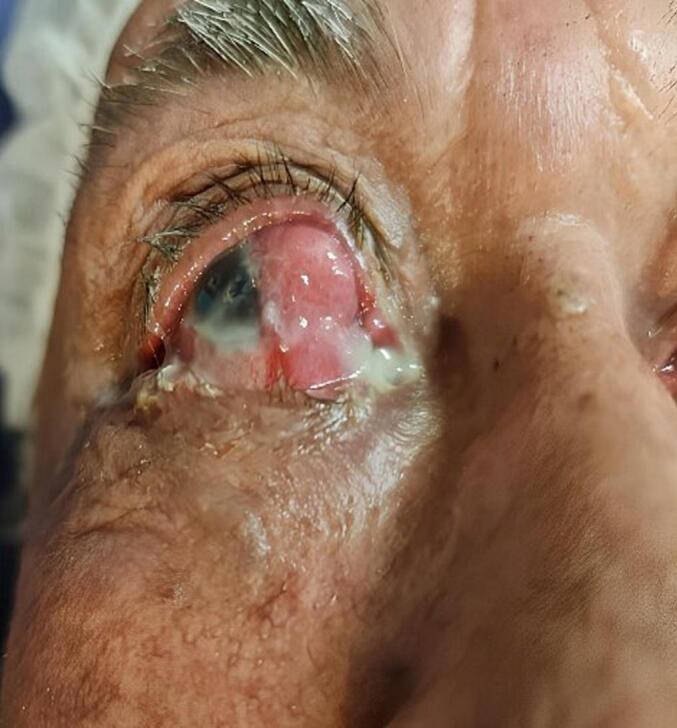
Solitary sessile lesion (16∗ 10∗ 7 mm) in diameter at the right nasal limbal region at the initial clinical examination.

The patient was an 83-year-old farmer man who presented with a lesion in the nasal part of his right eye six months before admission. The patient did not have any significant past medical, surgical, family, social (smoking or alcohol), or allergic history. Three months before the current admission the patient had another visit with an ophthalmologist and was misdiagnosed with pterygium. During this period the lesion progressed rapidly and this time with a giant lesion the patient was referred to our hospital and this time a thorough workup was done based on his condition. Examination revealed a solitary sessile lesion (16 ∗ 10 ∗ 7 mm) in diameter containing more than 6 h of the right nasal limbal region, categorized as a large lesion. It was mobile, non-tender, fleshy red in characteristic without any pigmentation. Vascularization and a feathery border of the lesion were noticeable (Fig. 1).

Anterior segment OCT (SPECTRALIS, Heidelberg Engineering, Germany) and ultrasound biomicroscopy (UBM) (ELLEX Eyecubed, Germany) were performed, and no signs of intraocular or adjacent tissue invasion were observed. All these findings were in favor of conjunctival squamous cell carcinoma. As immunocompromised status can be a risk factor for developing conjunctival SCC, the HIV antibody test was checked for the patient which was reported negative. Also, human papillomavirus PCR was checked and that was negative too. The lesion was removed via excisional biopsy using the “No Touch” technique to minimize the risk of tumor seeding. Although achieving tumor-free margins of at least 4 mm is ideal, due to the large size of the tumor, 90 % was excised during surgery. The conjunctival tumor was excised using Vannas scissors through a wide excisional biopsy. Topical anesthesia was administered with 5 drops of 0.4 % oxybuprocaine hydrochloride, followed by subconjunctival anesthesia using 2 % mepivacaine.

**Fig. 2:**
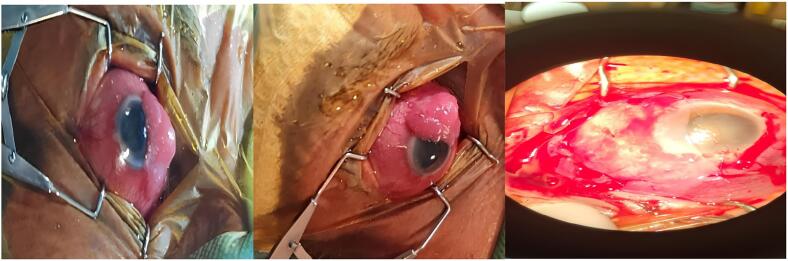
Lesion removal using the “No touch” technique: followed by amniotic membrane graft insertion.

According to the size of the wound defect, the primary closure of the conjunctiva was not accomplished, and because the bare sclera was considered a complication, an amniotic membrane graft was performed, and Topical 0.002 % Mitomycin C (Alte Apotheke, Stuttgart, Germany) was also used as a topical chemotherapy for 3 min, without the use of cauterization. The remaining 10 % of the lesion was completely resolved with topical Interferon alpha-2a (Essex Pharma, Luzern, Switzerland) at a concentration of 1 MIU/cc, applied four times daily for three months (Fig. 2).

**Fig. 3:**
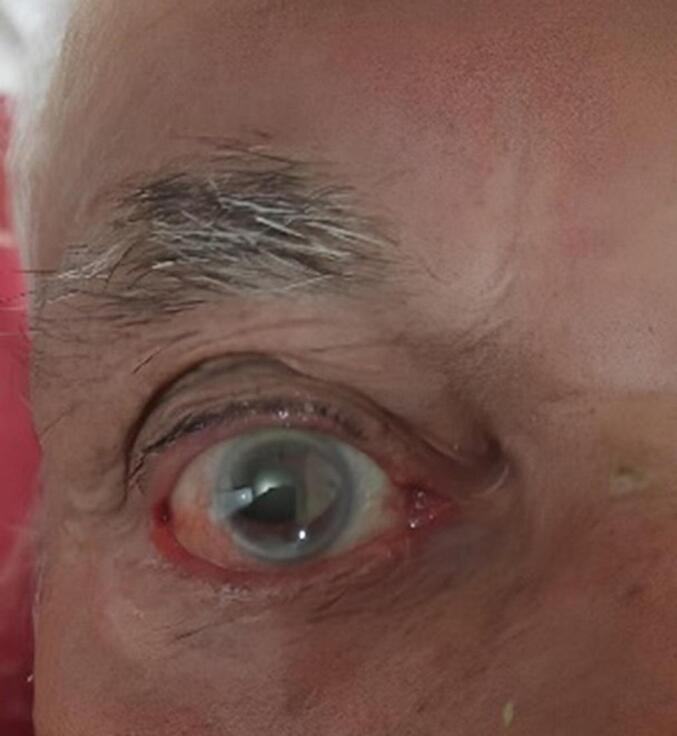
Final examination after 1 year without any recurrence of conjunctival squamous cell carcinoma.

We followed up with the patient at 1 month, 3 months, 6 months, and 1 year. No recurrence was observed at any of these intervals, and the patient is now tumor-free (Fig. 3).

## 3 Discussion

Conjunctival squamous cell carcinoma is the final stage of ocular surface squamous neoplasia (OSSN), with incidence varying by region [6]. Squamous cell carcinoma (SCC) is a treatable cancer, but misdiagnosis can delay appropriate treatment. This delay may result in disease progression, potentially advancing to a life-threatening stage if not promptly addressed [7].

Different risk factors were reported for this neoplasia such as older age, being male, white race, working outdoors (exposure to UV radiation), HIV/AIDS, and human papillomavirus [3,8]. In our patient, an elderly Caucasian male farmer with significant exposure to UV radiation, conjunctival squamous cell carcinoma was diagnosed.

Early-stage squamous cell carcinoma (SCC) can be managed with various treatments, including Interferon α-2b, Mitomycin C, and 5-Fluorouracil [9]. Observation is typically preferred for benign, asymptomatic tumors, with close follow-up every 6 to 12 months to monitor for dysplastic changes, growth, or effects on adjacent tissues [10]. For extensive or symptomatic tumors, incisional biopsy aids in treatment planning and may guide systemic therapy. Excisional biopsy is favored to prevent tumor seeding, particularly for smaller lesions [11]. In our patient, an excisional biopsy was performed due to the large size of the tumor. However, 10 % of the lesion remained and was successfully treated with topical Interferon alpha-2a (1 MIU/cc).

Numerous studies have emphasized the importance of chemotherapy in the management of ocular surface squamous neoplasia. Following resection, topical Mitomycin C (MMC) is recognized as a cost-effective and safe method to reduce tumor recurrence [12,13]. In our study, Mitomycin C was applied at a concentration of 0.002 % for 3 min before being washed off.

The bare sclera technique is one method that facilitates re-epithelialization; however, other techniques such as primary closure [1], autologous conjunctival graft and amniotic membrane graft are also available [14]. One study indicates that using an amniotic membrane graft not only achieves complete tumor resolution but also reduces the surgical burden, resulting in favorable functional and cosmetic outcomes.

Recurrences following surgical treatment of squamous cell carcinoma (SCC) are common [15]. with rates varying between 5 % and 56 % depending on the study and follow-up duration [16,17]. In our patient, despite the large size of the lesion and the inability to achieve margin-free excision, the residual tumor was successfully resolved with topical Interferon alpha-2a therapy. After a 1-year follow-up, all signs of the tumor had disappeared.

Follow-up is crucial for monitoring recurrence, as most recurrences typically occur within 3 to 6 months [1]. We monitored the patient at 1 month, 3 months, 6 months, and 1 year. No recurrences were observed at any follow-up visit, and the patient is now tumor-free.

## 4 Conclusion

This case highlights the successful management of advanced conjunctival squamous cell carcinoma through a combination of excisional biopsy, topical Interferon alpha-2a, and amniotic membrane grafting. The approach effectively resolved the residual tumor, prevented recurrence, and ensured favorable outcomes, demonstrating a comprehensive strategy for complex cases.

## Author contribution

Amirmohammad Fathian: study concept or design, data collection, data analysis or interpretation, writing the paper.

Navid Fazlinejad: study concept or design, data collection, data analysis or interpretation.

Alireza Attar: study concept or design, data collection, data analysis or interpretation, writing the paper.

## Consent to participate

Consent for publication (According to ICMJE Recommendations for protection of research participants).

## Consent

Written informed consent was obtained from the patient for publication of this case report and accompanying images. A copy of the written consent is available for review by the Editor-in-Chief of this journal on request.

## Ethical approval

According to research ethics committees of Shiraz university of medical sciences this study is exempt from ethical approval.

## Guarantor

Alireza Attar.

## Research registration number

Not applicable.

## Funding

None.

## Conflict of interest statement

No relevant conflict of interest.
